# Attack–Defense Game Model with Multi-Type Attackers Considering Information Dilemma

**DOI:** 10.3390/e25010057

**Published:** 2022-12-28

**Authors:** Gaoxin Qi, Jichao Li, Chi Xu, Gang Chen, Kewei Yang

**Affiliations:** College of Systems Engineering, National University of Defense Technology, Changsha 410073, China

**Keywords:** infrastructure networks protection, link hiding rule, Bayesian game, Harsanyi transformation

## Abstract

Today, people rely heavily on infrastructure networks. Attacks on infrastructure networks can lead to significant property damage and production stagnation. The game theory provides a suitable theoretical framework for solving the problem of infrastructure protection. Existing models consider only the beneficial effects that the defender obtains from information gaps. If the attacker’s countermeasures are ignored, the defender will become passive. Herein, we consider that a proficient attacker with a probability in the game can fill information gaps in the network. First, we introduce the link-hiding rule and the information dilemma. Second, based on the Bayesian static game model, we establish an attack–defense game model with multiple types of attackers. In the game model, we consider resource-consistent and different types of distributions of the attacker. Then, we introduce the solution method of our model by combining the Harsanyi transformation and the bi-matrix game. Finally, we conduct experiments using a scale-free network. The result shows that the defender can be benefited by hiding some links when facing a normal attacker or by estimating the distribution of the attacker correctly. The defender will experience a loss if it ignores the proficient attacker or misestimates the distribution.

## 1. Introduction

Critical infrastructures, such as electrical power systems, communications systems, and oil pipeline systems, exist in the form of networks and play an essential role in the lives of modern residents. Damage to these infrastructures brings tremendous economic losses and generates negative social influence. The importance of infrastructure attracts terrorists and enemies during wars. Recently, the Crimean Bridge, which undertook a resupply mission for the Russian army, was blown up, and one-third of the Ukrainian power plants were destroyed, leading to power outages across the country. The protection of infrastructure networks must be settled urgently.

Most research mainly considers the antagonism between the attacker and the defender and studies the allocation of defensive resources or the establishment of defensive strategies by building a game model suitable for the characteristics of various infrastructures. Feng et al. [1] established a static game model and Bayesian game model to analyze defense allocation for chemical facilities. Regarding the power system, Tas et al. [2] considered the cascade failure of the power grid and analyzed how the attacker harnesses it in the game. For transposition systems, Talarico et al. [3] built a framework to warn against impending attacks on the transportation infrastructure.

Combining game theory with complex networks theory, Li et al. [4,5,6] considered different disintegration strategies and analyzed the influence of network structure on equilibrium. Zeng et al. [7] contracted a false network to mislead an attacker by reconnecting links and studied the influence of asymmetric information on the game. They also built a Bayesian game model to solve the problem of multi-type attackers who have different payoff functions [8]. We design a link hiding rule to create the information gap between the defender and the attacker and compare the benefit of hiding links with reconnected links [9].

In previous research, there are some methods that build information gaps to mislead the attacker in game, such as hidden node information, hidden links in networks, and the construction of false links. However, the attackers can fill those gaps through reconnaissance and link predictions. In this paper, we build a Bayesian game model with a defender and an attacker to study the situation in which hidden links are discovered. We assume that there exist several types of attacker. The proficient attacker can discover the whole network structure, and the normal attacker cannot find the hidden links. In the view of the defender, the different type of attacker exists with a distribution of probability, which is prior probabilities in a Bayesian game. Then we calculate the Bayesian equilibrium in different parameter combinations. We consider the two situations of misjudgment. The result is shown that underestimating the probability of the proficient attacker is more serious.

The remainder of this paper is organized as follows: Section 2 introduces some related works; Section 3 introduces a link-hiding rule and information dilemma. Section 4 establishes an attack–defense game model based on the Bayesian static game model and presents the solution method; Section 5 shows the equilibrium results in a scale-free network and analyzes the impact of link information. Section 6 concludes this paper.

## 2. Related Work

Our study is related to the protection of infrastructure using game theory. Researchers have used different modeling methods and game models for different scenarios. A static game model is typically used to solve the problem of choice. The dynamic game model is suitable for situations in which offensive and defensive actions are in multiple phases and not simultaneous. A Bayesian game model is built to resolve the uncertainty problem, which can be estimated using probability distributions.

Using a static game model, Bier et al. [10] studied the reallocation of attack and defense sources. Feng et al. [1] studied how to optimize the allocation of defensive resources for multiple chemical facilities. They considered the influence of chemical materials when chemical facilities were attacked and used this influence as the measure function. Baykal-Guersoy et al. [11] considered the number of people affected or the occupancy level of critical infrastructure as a risk measure after attacking the infrastructure security game. Chen et al. [12] evaluated the performance of defense strategies using a two-person, zero-sum game model. Fu et al. [13] developed a two-person static game model for the cascade effect of the infrastructure and analyzed a pure and mixed strategy equilibrium. Li et al. [4] used the largest connected component of a network as a metric function and investigated the effect of the network structure on the equilibrium solution.

Using a dynamic game model, Baykal-Guersoy et al. [11] studied the protection of critical infrastructure in multiple stages. Brown et al. [14] established a defender–attacker model and a defender–attacker–defender game model to study homeland defense, which is a multiple-phase game. Li et al. [6] investigated the effect of the first-mover advantage on equilibrium. Fu et al. [15] first protected the network through protective or camouflaged behavior.

Using a Bayesian game model, Zhang et al. [16] classified an attacker into two types using different cost methods and analyzed how to choose defense strategies using the Bayesian Nash equilibrium. Zeng et al. [8] built a two-type attacker game model in which different attackers have different payoff functions. Feng et al. [17] studied a game for chemical facilities with multiple types of attackers, and different chemical facilities had different values for different types of attackers. Jiang et al. [18] developed a Bayesian Stackelberg game model to study the problem of water supply network protection, including four private information cases. Gu et al. [19] built a Bayesian Stackelberg game model for attackers with different utility functions and analyzed the effect of the type of distribution on the equilibrium solution.

Much research has been conducted to protect infrastructure in various scenarios using game theory. However, they ignored the situation of hiding the information being found. We proposed a link-hiding rule in a previous work, which can build an information gap in the network structure. We study the situation in which an attacker discovers hidden structural information and how to deal with it.

## 3. Link Hiding Rule and Information Dilemma

In this section, we introduce a link-hiding rule whose validity is proven in dynamic games. Furthermore, we consider the situation in which hidden links are discovered and analyze why the defender is influenced by the situation.

### 3.1. Link Hiding Rule

The link is an essential part of the network, representing various relationships between nodes, and plays the roles of transmission, transportation, and transformation. The importance of the links and nodes is interrelated. For example, when a node has more links, its degree centrality is high. Simultaneously, a link connecting two nodes with higher degrees is more important. There are several ways to change a network’s structure, such as reconnecting links [20] and adding links [21]. To reduce the damage to principal targets by attackers as much as possible, we assume that the probability of a hidden link connection is positively related to the properties of the nodes on both sides of the link. The number of hidden links depended on the network structure.

Infrastructure networks can be presented as a simple undirected graph G=(V,E), where $V=[v_{1},v_{2},\cdots ,v_{N}]$ represents the node set, and $E={\left(e_{ij}\right)}_{M}\subseteq V\times V$ represents the link set. The number of nodes and links are N=|V| and M=|E|, respectively. Let $A\left(G\right)={\left(a_{ij}\right)}_{N\times N}$ represent the adjacency matrix of graph *G*. $a_{ij}=a_{ji}=1$ if a link exists between $v_{i}$ and $v_{j}$; otherwise, $a_{ij}=a_{ji}=0$. Let $r_{i}>0$ represent the properties of nodes, for example, the degree, betweenness, or capability of nodes. Sorting ${\left\{r_{i}\right\}}_{N}$ in the descending order, we obtain $r_{\left(1\right)}\geq r_{\left(2\right)}\geq \cdots \geq r_{\left(N\right)}$. Let $k_{i}$ represent the degree of the node $v_{i}$. Then the weighted average of $r_{i}$ can be defined as $\bar{r}=\frac{\sum_{i=1}^{N}k_{i}r_{i}}{\sum_{i=1}^{N}k_{i}}$.

We design the sum of hidden links as αM and define the hidden probability of the link as $p_{ij}$ associated with the property of node $v_{i}$ and $v_{j}$, then $p_{ij}$ can be represented as:

(1) $$p_{ij}=\alpha M\frac{r_{i}+r_{j}}{\sum_{i=1}^{N}k_{i}r_{i}}$$

where $\alpha \in [0,\frac{2}{r_{\left(1\right)}+r_{\left(2\right)}}\bar{r}]$ is called the average hiding coefficient. $\sum p_{ij}=\alpha M$.

### 3.2. Information Dilemma

**Figure 1:**
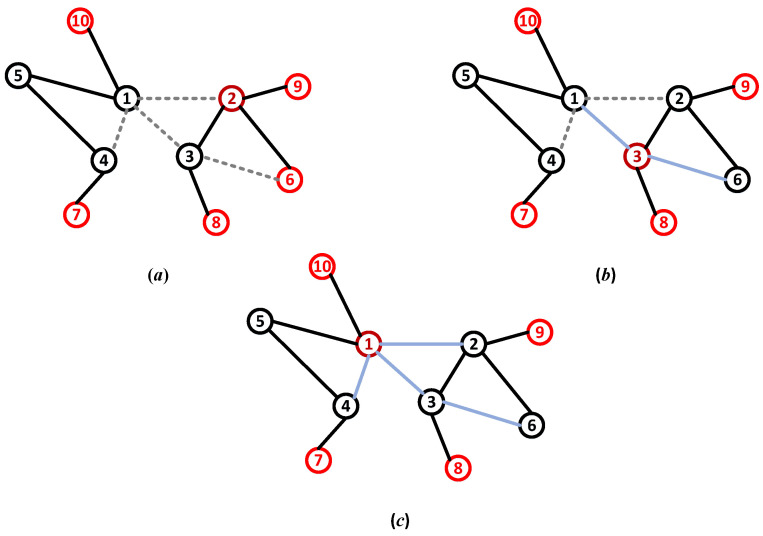
The networks structure information held by different types of attackers, (**a**) normal attacker, (**b**) semi-proficient attacker, (**c**) proficient attacker. The gray dotted line represents the hidden link, and the blue line represents the hidden link discovered by the attacker.

In cities, communication and power cables exist in the form of burial [22]. This creates the conditions for hiding some of the links. The defender can mislead the attacker’s strategy choices by hiding a part of the link. For example, from the attacker’s perspective, the node’s highest degree is 2 in Figure 1a after hiding links, and the lowest degree of nodes is 6, 7, 8, 9, and 10. This value deviates from the actual value. In addition, it is assumed that a node with five degrees is destroyed, requiring five units of offensive recourse. Then, Nodes 2 and 6 are attacked with unsaturated resources, which may lead to attack failure. Hiding links is not always effective. An attacker can find hidden links by scouting or link prediction. As shown in the figure, the attack strategies vary with different network topology information. Attackers are classified based on their level of information. We refer to the type of attacker in Figure 1a as the normal type, the type in Figure 1b as the semi-proficient type, and the type in Figure 1c as the proficient type. The network after hiding links is called a misleading network.

The link hiding creates a dilemma for the defender facing multi-type attackers. On the one hand, link hiding can benefit the defender by misleading the normal attacker. On the other hand, link hiding will bring loss to the defender who does not effectively deal with the proficient attacker. The dilemma is built by the information on network structure. To defuse the attack, the defender adapts the optimal reaction strategy to the attack strategy as the defense strategy. The attack strategies change with the attacker’s topology information, which is uncertain to the defender.

## 4. The Attack–Defense Game Model with Multi-Type Attackers under Information Dilemma

Considering the information dilemma caused by hidden links, we built a static game model based on Bayesian games. The type of attacker is uncertain, but the defender can estimate the distribution of the type. To simplify the model, we consider the proficient and normal types of attacker, and the distribution is estimated to Ω=(ω,1−ω).

### 4.1. Cost Model

We considered only the strategy of the node here. When a node is removed, the links are removed. The source of the attack and defense node $v_{i}$ is denoted by $c_{i}$, a parameter related to the node’s property $r_{i}$:

(2) $$c_{i}=r_{i},$$

Assuming that the resources of attack or defense for all nodes are $\tilde{C}$, then:

(3) $$\tilde{C}=\sum_{i=1}^{N}c_{i}=\sum_{i=1}^{N}r_{i},$$

we donate $\theta^{A}\in \left[0,1\right]$ as the cost constraint coefficient, the attacker’s available resources can be represented as ${\tilde{T}}^{A}=\theta^{A}{\tilde{C}}^{A}=\theta^{A}\sum_{i=1}^{N}r_{i}^{\beta }$. Similarly, we can define ${\tilde{C}}^{D}$ and ${\tilde{T}}^{D}$ by ${\tilde{C}}^{D}=\sum_{i=1}^{N}c_{i}^{D}=\sum_{i=1}^{N}\left(r_{i}\right)$, and ${\tilde{T}}^{D}=\theta^{D}{\tilde{C}}^{D}=\theta^{D}\sum_{i=1}^{N}r_{i}$, respectively.

The $d=\left(d_{1},d_{2},\cdots ,d_{N}\right)\in S_{D}$ is donated as a defensive strategy satisfying resource constraints, where $S_{D}$ is the strategy set of the defender. If the node is defended, we have $d_{i}=1$; otherwise, $d_{i}=0$. The cost of *d* is:

(4) $$C_{d}^{D}=\sum_{v_{i}\in V^{D}}r_{i}=\sum_{i=1}^{N}d_{i}r_{i}\leq {\tilde{T}}^{D}=\theta^{D}\sum_{i=1}^{N}r_{i}.$$

Similarly, we can define the attacker’s cost $C_{a}^{A}$, where $a=\left[a_{1},a_{2},\ldots ,a_{N}\right]\in S_{A}$:

(5) $$C_{a}^{A}=\sum_{v_{i}\in V^{A}}r_{i}=\sum_{i=1}^{N}a_{i}r_{i}\leq {\tilde{T}}^{A}=\theta^{A}\sum_{i=1}^{N}r_{i}.$$

We note that $r_{i}$ of the same nodes might be different for the attacker and defender. When links are hidden, $r_{i}$ is changed in the infrastructure networks, which means a cost change. Insufficient attacks may not damage the nodes. Let $V^{A}$ represent the set of attacked nodes. We define the success rates to reflect this effect: $\forall v_{i}\in V^{A}$

(6) $$ps_{i}=\left\{\begin{array}{cc} 0 & \;\mathrm{if}\;\frac{c_{i}^{A}-c_{i}^{D}}{r_{i}}<0\\ \frac{c_{i}^{A}-c_{i}^{D}}{r_{i}} & \;\mathrm{if}\;0\leq \frac{c_{i}^{A}-c_{i}^{D}}{r_{i}}\leq 1\\ 1 & \;\mathrm{if}\;1<\frac{c_{i}^{A}-c_{i}^{D}}{r_{i}}. \end{array}\right.$$

where $r_{i}^{A}$ and $r_{i}^{D}$ represent the node properties in the views of the attacker and defender, respectively. Here, $r_{i}$ is the degree of the nodes.

### 4.2. Strategy Set

Here, we only consider two typical strategies:

(1) High-degree attack or defense strategy. A high-degree attack strategy damages nodes with a high degree. The high-degree defense strategy defends nodes with a high degree.
(2) Low-degree attack or defense strategy. The low-degree attack strategy is aimed at nodes with a low degree. Because the resources consumed are relatively low compared with high-degree nodes, the low-degree attack at the same cost can destroy more low-degree nodes. The low-degree defense strategy defends nodes with a low degree.

Specifically, the attacker develops strategies based on the network structure it owns. A proficient attacker adopts a strategy based on the true network, and thus it has a high-degree attack strategy in true networks (THA) and a low-degree attack strategy in true networks (TLA). A normal attacker adopts a high-degree attack strategy in misleading networks (MHA) and a low-degree attack strategy in misleading networks (MLA). As the best response strategies, defenders need to consider four strategies: high-degree defense strategy in true networks (THD), low-degree defense strategy in true networks (TLD), high-degree defense strategy in misleading networks (MHD), and low-degree defense strategy in misleading networks (MLD).

### 4.3. Payoff Function

We denote the measure function of the network performance by Γ, including the efficiency and size of the largest connected component. Let $\hat{G}$ represent the network after a game round. Then, the defender’s payoff function is

(7) $$u^{D}\left(a,d\right)=\frac{\Gamma \left(\hat{G}\right)-\Gamma \left(G\right)}{\Gamma (G)}.$$

Similarly, the payoff of the attacker can be defined as:

(8) $$u^{A}\left(a,d\right)=\frac{\Gamma \left(G\right)-\Gamma \left(\hat{G}\right)}{\Gamma (G)}.$$

Here the *G* might be different between Equations (7) and (8) for partly hiding links.

## 5. Solution Method

The Bayesian Nash equilibrium is a general solution for the Bayesian game model, and we used it as the solution here. The solution form is ((the equilibrium of type 1 of the attacker, the equilibrium of type 2 of the attacker), the equilibrium of the defender). Let the distributions of the defender’s strategy and the attacker’s strategy be $P=(p_{1},p_{2},\cdots ,p_{n}{)}^{\prime }$ and $Q_{k}={\left(q_{1}^{a_{k}},q_{2}^{a_{k}},\ldots ,q_{m^{a_{k}}}^{a_{k}}\right)}^{\prime }\in {\left[0,1\right]}^{m^{a_{k}}}$, where *n* and $m^{a_{k}}$ represent the number of defender’s and k−th type of attacker‘s strategies, respectively. The attacker’s objective function can be donated as:

(9) $$\max O^{a_{k}}=\max P^{\prime }U^{a_{k}}Q_{k},$$

where $U^{a_{k}}$ represents the payoff matrix of the k−th type of attacker.

The defender’s objective function can be denoted as follows:

(10) $$\max O^{d}=\max\sum_{k=1}^{K}\omega_{k}P^{\prime }U^{d}Q_{k},$$

where $U^{d}$ represents the payoff matrix of the defender.

The attack–defense game model established here is a Bayesian static game model in which both attackers and defenders act simultaneously. In other words, before the game occurs, neither the attacker nor the defender knows which strategy the other side has adopted. The Bayesian static game model can be transformed into a complete information static game model using Harsanyi transformation [23]. The solution of the Bayesian static game model is defined as the Bayesian Nash equilibrium (BNE). Let the solution be of the form $\left(P^{*},Q_{1}^{*},Q_{2}^{*},\ldots ,Q_{K}^{*}\right)$ Then, we have:

(11) $${P^{*}}^{\prime }U^{a_{k}}Q_{k}^{*}\geq {P^{*}}^{\prime }U^{a_{k}}Q_{k}$$

(12) $$\sum_{k=1}^{K}\omega_{k}{P^{*}}^{\prime }U^{a_{k}}Q_{k}^{*}\geq \sum_{k=1}^{K}\omega_{k}P^{\prime }U^{a_{k}}Q_{k}^{*}$$

Specifically, we considered a payoff matrix for two types of attackers. When the attacker is normal, the attacker does not have full information about the network structure. At this point, it creates strategies and calculates the payoff according to the misleading network. When the defender adopts THD and TLD, the attacker views them as MHD and MLD to calculate the payoff. When the attacker is proficient, it has full information about the entire game and knows all the strategies the defender may adopt. The payoff at this time is the value both parties calculated based on the real network. Then we can calculate the payoff matrices based on payoff function, and payoff matrices of proficient and normal attacker are shown in Table 1 and Table 2, respectively.

Based on the Harsanyi model [24,25], we can turn it to Table 3. We can solve this bi-matrix game using linear programming [26].

## 6. Experiments

Most real-world networks are scale-free, such as airplane networks [27] and bank payoff networks [28]. We used the Barabási–Albert model to construct a scale-free network with 300 nodes and an average degree of 2. We conducted 500 independent experiments based on this network and obtained the average payoff. Main definitions used in this section are shown in Table 4.

### 6.1. Benefits of the Link Hiding for the Defender

**Figure 2:**
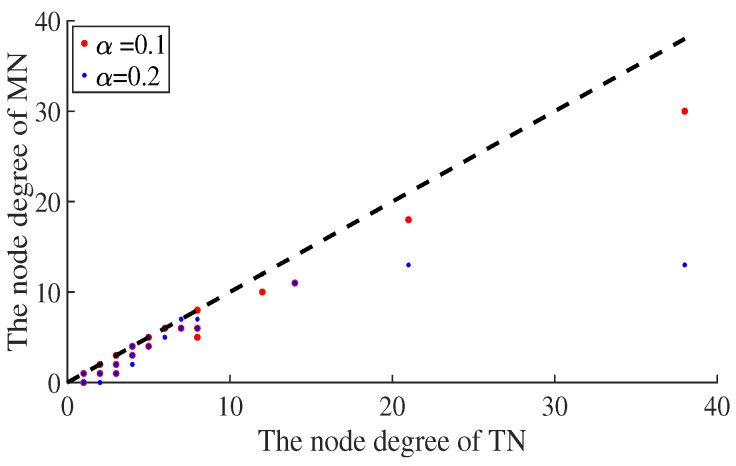
The degree value of the node in the true network (TN) and the misleading network (MN). The x-axis represents the true degree value of the node, and the y-axis represents the degree value of the node behind the hidden link. The red and blue dots represent the change in the degree value of the node when the link hidden coefficient α=0.1 and α=0.2, respectively.

The degree values of the nodes with different link hidden coefficients are shown in Figure 2. According to the figure, the height degree of the node decreases more than the low degree of the nodes, with an increase in the average link hiding coefficient. Hiding some links disturbs the order of the degrees. The high-degree attack strategy based on a misleading network excludes some nodes, although its degree is high in reality.

**Figure 3:**
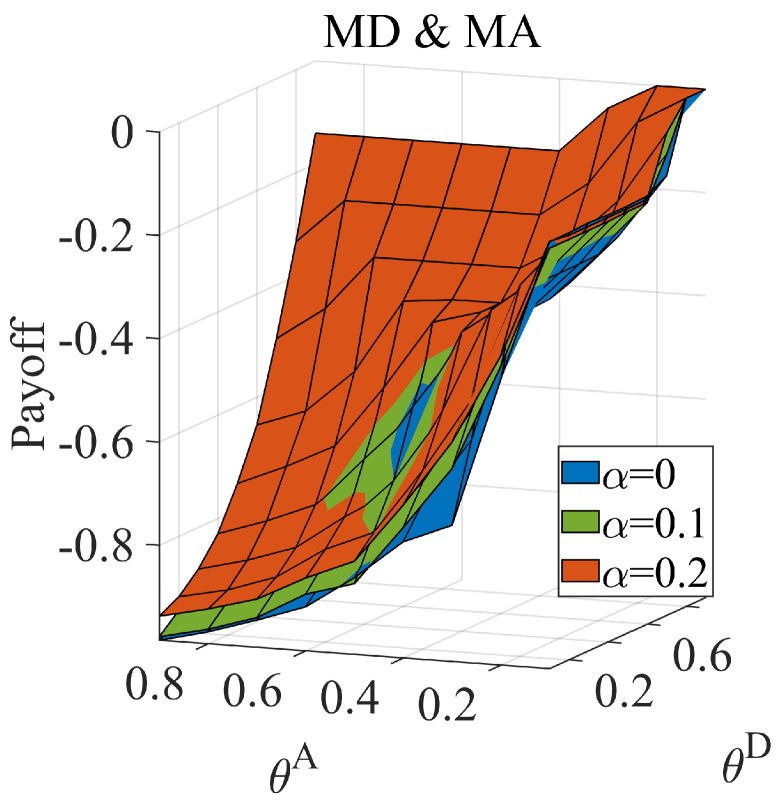
The defender’s equilibrium payoff when the defender adopts the MD set facing the normal attacker, when attack cost constraint coefficient $\theta^{A}\in \left[0.1,0.9\right]$, defense cost constraint coefficient $\theta^{D}\in \left[0.1,0.9\right]$, average link hiding coefficient α=0,0.1,0.2.

If the link hiding cannot improve the defender’s payoff in any situation, an information dilemma does not exist. We prove the benefit to the defender facing the normal attacker by partly hiding links. Facing a normal attacker, ω equals zero, and the game degenerates into a complete information game. We calculate the payoff of the Nash equilibrium when applying MD (the strategy set contains MHD and MLD) against MA (the strategy set contains MHA and MLA), and the results are shown in Figure 3. We found that hidden links can benefit the defender and that the benefit to the defender increases with an increase in the link hidden coefficient.

**Figure 4:**
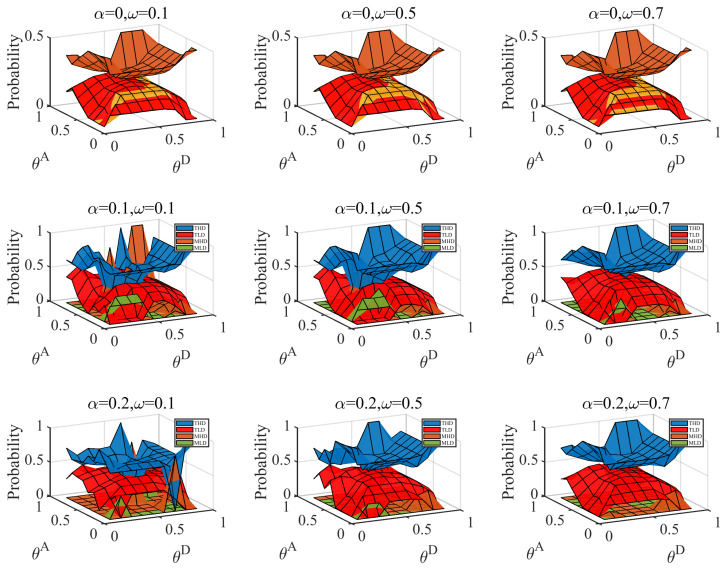
Probabilities of the defender’s strategies in Bayesian Nash equilibrium under different attack cost constraint coefficient $\theta^{A}$ and defense cost constraint coefficient combinations, when average link hiding coefficient α=0,0.1,0.2, probability of the proficient attacker ω equal 0.1,0.5,0.7. The yellow grid represents when TLD and MLD coincide.

**Figure 5:**
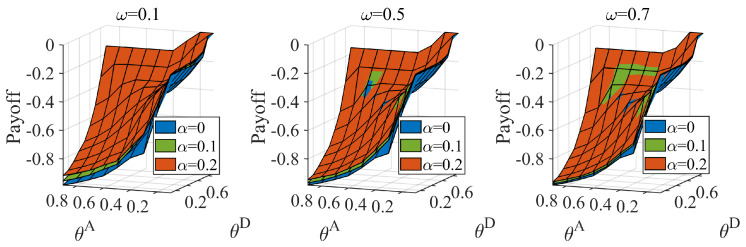
Defender’s equilibrium payoff under different attack cost constraint coefficient $\theta^{A}$ and defense cost constraint coefficient $\theta^{D}$ combinations, when average link hiding coefficient α=0,0.1,0.2, probability of the proficient attacker ω equal 0.1,0.5,0.7.

We computed the Bayesian Nash equilibrium of the defender when the distributions of the attacker type Ω=(ω,1−ω) were (0.1,0.9), (0.5,0.5), (0.7,0.3). The results are shown in Figure 4. According to the figure, the probability of the defender adopting the real strategy gradually increases with a gradual increase in the number of hidden links and the probability of a highly proficient attacker. We observed that when the probability of a proficient attacker is low, the defender still adopts TD (the defense strategies based on the true network) in some cases, particularly THD (the high-degree defense strategy based on the true network). TD covers more real critical nodes and allocates more resources to protect the network. The defender adopts MD (the defense strategies based on a misleading network), which can better cope with the normal attacker when the defender’s cost constraint coefficient and the probability of the proficient attacker are both low. The MD cost is lower than TD. We also calculated the defender’s equilibrium payoff; the results are shown in Figure 5. As seen in Figure 5, the defender’s payoff increases as the defense cost increases and the attack cost decreases. Hiding partial links can benefit the defender in most situations in the Bayesian Nash equilibrium. There are also singularities when the probability of a proficient attacker is high.

### 6.2. Equilibrium with Different Distributions of the Attacker’s Type

**Figure 6:**
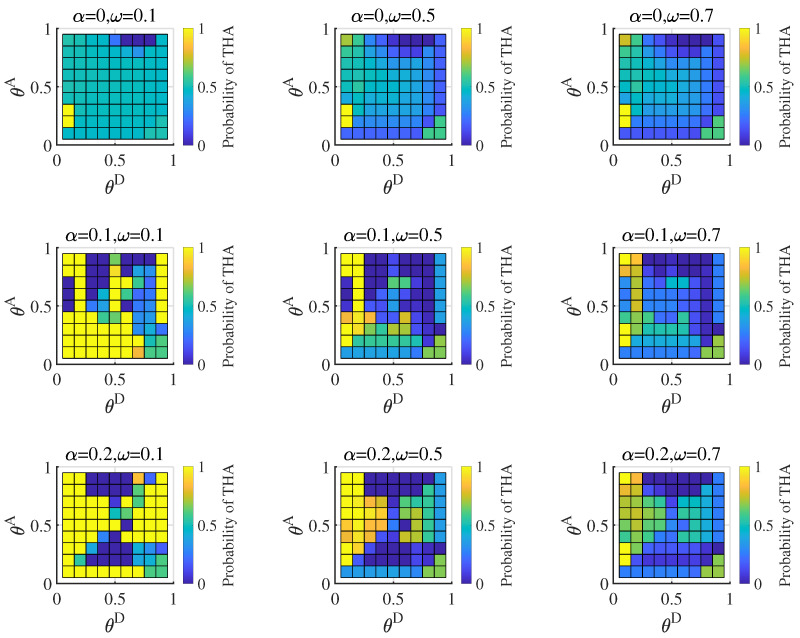
Probability of the proficient attacker adopting high-degree attack strategies based on the true network under different attack cost constraint coefficient ($\theta^{A}\in \left[0.1,0.9\right]$) and defense cost constraint coefficient ($\theta^{D}\in \left[0.1,0.9\right]$) combinations in Bayesian Nash equilibrium, when average hiding coefficient α=0, 0.1, 0.2, probability of the proficient attacker ω=0.1, 0.5, 0.7.

The attacker is the proficient type, and its equilibrium strategy is shown in Figure 6. When the attacker has more resources than the defender, it adopts the high-degree strategy to attack the critical nodes, which cannot be covered by the high-degree defense strategy for the low-defense resource. When the defender estimates the probability of a proficient attacker as low, the defender mainly copes with the attack strategy based on the misleading network. Therefore, the proficient attacker will likely use high-degree strategies, which can obtain more payoff for destroying critical nodes. With the increase in the average hiding coefficient, the gap between the strategy based on the true network and the strategy based on the misleading network increases. The proficient attacker obtains more payoff by increasing the probability of the high-degree attack strategy. When the defender estimates the probability of a proficient attacker increase, the defender mainly resists the attack strategies based on the true network. The defender protects the critical nodes with a high-degree defense strategy. The proficient attacker decreases the probability of the high-degree strategy to avoid defense.

**Figure 7:**
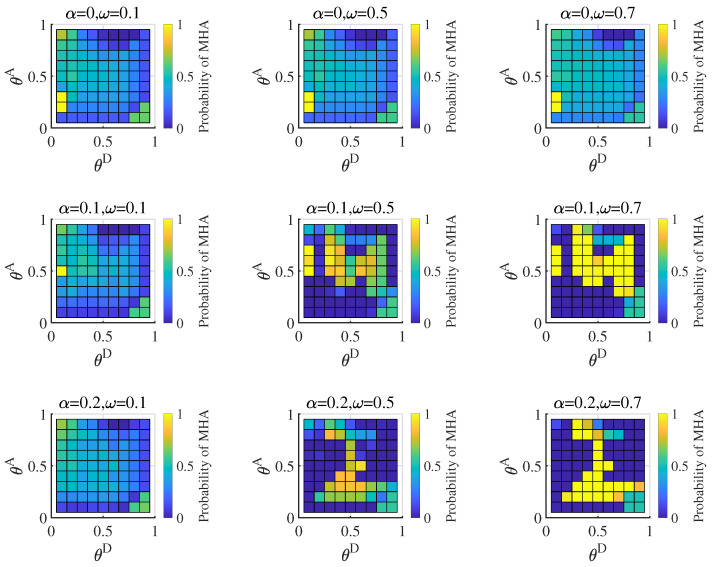
Probability of the normal attacker adopting MHA (the high-degree attack strategy based on the misleading network) in Bayesian Nash equilibrium under different attack cost constraint coefficient ($\theta^{A}\in \left[0.1,0.9\right]$) and defense cost constraint coefficient ($\theta^{D}\in \left[0.1,0.9\right]$) combinations, when average hiding coefficient α=0,0.1,0.2, probability of the normal attacker 1−ω=0.9,0.5,0.3.

When the attacker is of the normal type, the equilibrium strategy is shown in Figure 7. The normal attacker has a similar result to the proficient attacker. When the probability of the normal type is high (1−ω=0.9), the defender pays more attention to strategies based on the misleading network. Link hiding has almost no effect on the equilibrium at this time. With the decrease in the probability of the normal type, attack strategies based on the misleading network cannot be held back, and the normal attacker adopts the pure strategies. However, our calculation of the equilibrium payoff is insignificant. A normal attacker may not adopt the strategy distribution we provided. According to the Nash equilibrium definition, when the attacker adopts other strategy distributions, the payoff obtained is less than or equal to the equilibrium payoff.

### 6.3. Influence of the Misjudgment on the Defender

**Figure 8:**
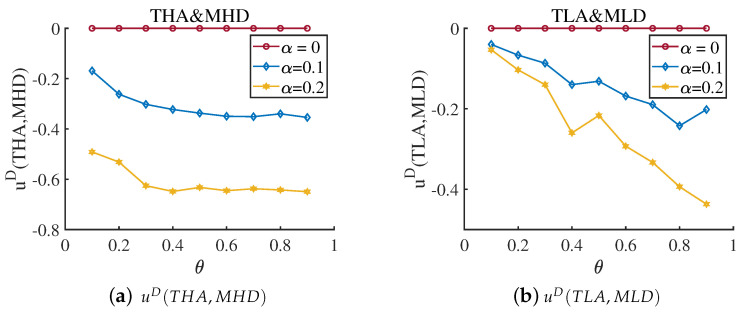
Payoff $u^{D}\left(THA,MHD\right)$ and payoff $u^{D}\left(TLA,MLD\right)$ when the average hiding coefficient α=0,0.1,0.2 and cost coefficient $\theta =\theta^{A}=\theta^{D}\in \left[0.1,0.9\right]$. The THA represents the high-degree attack strategy based on the true network, and the TLA represents the low-degree attack strategy based on the true network. The MHD represents the high-degree defense strategy based on the misleading network, and the MLD represents the low-degree defense strategy based on the misleading network. The Subfigure (**a**) reflect the gap between THA and MHD, and the Subfigure (**b**) reflect the gap between TLA and MLD. Gaps increases when the α increases, which means the defender cannot cope with the attacker although it chooses right defense mode.

**Figure 9:**
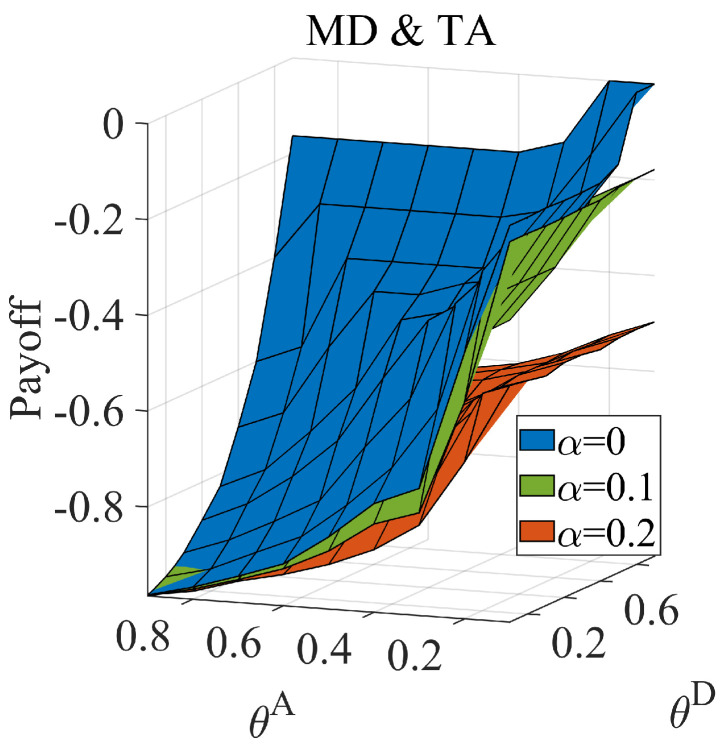
Defender’s equilibrium payoff when the defender adopts MHD (high-degree defense strategy based on the misleading network) and MLD (low-degree defense strategy based on the misleading network) facing a proficient attacker under different attack cost constraint coefficient ($\theta^{A}\in \left[0.1,0.9\right]$) and defense cost constraint coefficient ($\theta^{D}\in \left[0.1,0.9\right]$) combinations.

Consider the first information dilemma, in which the defender ignores the existence of the proficient attacker. When the attacker is a proficient type, and the defender does not know it at all, we calculate $u^{D}\left(THA,MHD\right)$ and $u^{D}\left(TLA,MLD\right)$ when α=0,0.1,0.2 and $\theta =\theta^{A}=\theta^{D}$, and the result is shown in Figure 8. At this moment, the defender and attacker have the same cost constraint coefficient. The gap between the corresponding “optimal response strategy” gradually increases with the link hiding coefficient. We also calculate the equilibrium payoff for this situation. The results are shown in Figure 9. This information gap reduces the defender’s payoff.

**Figure 10:**
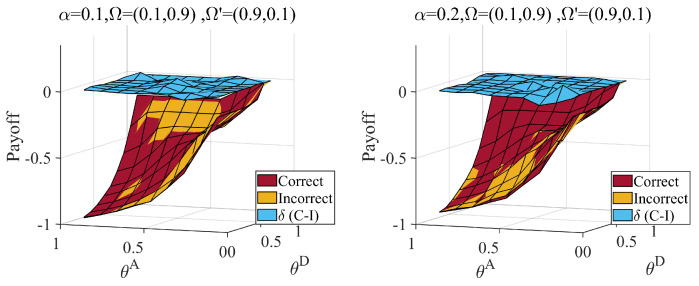
Defender’s payoff with correct (Ω=(0.1,0.9)) and incorrect ($\Omega^{\prime }=\left(0.9,0.1\right)$) judge attacker probability distributions, when attack cost constraint coefficient $\theta^{A}\in \left[0.1,0.9\right]$, defense cost constraint coefficient $\theta^{D}\in \left[0.1,0.9\right]$, average hiding coefficient α=0.1,0.2. The δ(C−I) represents the payoff difference between correct and incorrect situation. Link hiding brings about a loss to the defender when underestimating normal attackers.

**Figure 11:**
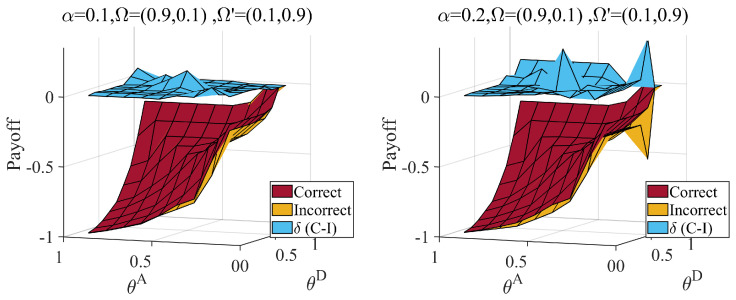
Defender’s payoff when correct (Ω=(0.9,0.1)) and incorrect ($\Omega^{\prime }=\left(0.1,0.9\right)$) judge attacker probability distributions, attack cost constraint coefficient $\theta^{A}\in \left[0.1,0.9\right]$, defense cost constraint coefficient $\theta^{D}\in \left[0.1,0.9\right]$, and average hiding coefficient α=0.1,0.2. The δ(C−I) represent the payoff difference between correct and incorrect situation. Link hiding brings about a loss to the defender when underestimating proficient attackers.

Second, we considered the information dilemma triggered by the misjudgment of the distribution of attacker types. The defender’s judgment of the type of attacker is also based on data experience and other methods. There is uncertainty in this method; therefore, there is a situation in which the attacker may be misled about the distribution. We calculate the payoff when the true type distribution is Ω=(0.1,0.9), the misjudgment distribution is $\Omega^{\prime }=\left(0.9,0.1\right)$, the true type distribution is Ω=(0.9,0.1) and the misjudgment distribution is $\Omega^{\prime }=\left(0.1,0.9\right)$. The results are shown in Figure 10 and Figure 11.

As shown in Figure 10, in the face of the first type of misjudgment, the defender’s payoff decreases in most cases, and the decrease in the payoff increases with an increase in the link hidden coefficient. There are also some singularities in this situation. When the average hiding coefficient α is equal to 0.1 and the defense cost coefficient $\theta^{D}$ is greater than 0.5, the payoff of incorrect judgment is greater than that of correct judgment for protecting critical nodes. With an increase in the average hiding coefficient, the degree of high-degree nodes decreases, which can be contained in a high-degree strategy based on misleading networks at a lower cost. Thus, when the average hiding coefficient α equals 0.2, the singularities appear at $\theta^{D}$ lower than 0.5.

From Figure 11, we can obtain similar conclusions when Ω=(0.9,0.1) and $\Omega^{\prime }=\left(0.1,0.9\right)$. An incorrect estimation can result in extra loss for hiding partial links. The loss increases with an increase in the average hiding coefficient. Combined with Figure 10 and Figure 11, the loss of the underestimated the probability of the proficient attacker is more than that of the underestimated normal attacker.

## 7. Conclusions

Technology has facilitated the construction of infrastructure networks and has brought great convenience to people’s lives, making residents increasingly dependent on them. To effectively protect the infrastructure network, it is necessary to combine game theory and complex network theory to study this problem. The link information plays important roles for the game participants as an essential part of infrastructure networks. The link hiding can benefit the defender facing normal attackers by building information gaps. However, when facing a proficient attacker, it causes trouble for the defender. We call the situation an information dilemma.

In this paper, we study the information dilemma by establishing a Bayesian game model. First, we introduce the link hiding rule, which is an effective method to build an information gap, and translate why the information dilemma exists. Second, we build a Bayesian game model with a multi-type of attacker. Then, we introduce the solution method. Finally, we experiment in a scale-free network. The result is shown that link hiding benefits the defender when facing the normal attacker. By the Bayesian Nash equilibrium, the defender copes with the different types of attacker and benefits by partly hiding links. We also analyze the situation of missed and incorrect judgments, which proves disadvantageous in link hiding. We should pay more attention to the proficient attacker.

Only two typical strategies are considered in our model, and more possible strategies will be considered in future work. In addition, the game model that we established mainly faces perfect rational participants. In reality, people are not always completely rational [29]. Therefore, the bounded rational groups should be studied in the future. We will make the game model more practical in the following work.

## Figures and Tables

**Table 1:** The payoff matrix of proficient attacker, whose probability is ω.

Type	Proficient Attacker (ω)	
Strategy	*THA*	*TLA*
THD	$u_{11}^{d_{1}},u_{11}^{a_{1}}$	$u_{12}^{d_{1}},u_{12}^{a_{1}}$
TLD	$u_{21}^{d_{1}},u_{21}^{a_{1}}$	$u_{22}^{d_{1}},u_{22}^{a_{1}}$
MHD	$u_{31}^{d_{1}},u_{31}^{a_{1}}$	$u_{32}^{d_{1}},u_{32}^{a_{1}}$
MLD	$u_{41}^{d_{1}},u_{41}^{a_{1}}$	$u_{42}^{d_{1}},u_{42}^{a_{1}}$

**Table 2:** The payoff matrix of normal attacker, whose probability is 1−ω.

Type	Normal Attacker (1−ω)	
Strategy	*MHA*	*MLA*
THD	$u_{11}^{d_{2}},u_{31}^{a_{2}}$	$u_{12}^{d_{2}},u_{32}^{a_{2}}$
TLD	$u_{21}^{d_{2}},u_{41}^{a_{2}}$	$u_{22}^{d_{2}},u_{42}^{a_{2}}$
MHD	$u_{31}^{d_{2}},u_{31}^{a_{2}}$	$u_{32}^{d_{2}},u_{32}^{a_{2}}$
MLD	$u_{41}^{d_{2}},u_{41}^{a_{2}}$	$u_{42}^{d_{2}},u_{42}^{a_{2}}$

**Table 3:** The payoff matrix after the Harsanyi transformation.

Strategy	THA, MHA	THA, MLA	TLA, MHA	TLA, MLA
THD	$\omega u^{d_{1}}+\left(1-\omega \right)u^{d_{2}},\omega u^{a_{1}}+\left(1-\omega \right)u^{a_{2}}$			
TLD				
MHD				
MLD				

**Table 4:** Abbreviations and definitions.

Abbreviations	Definitions
BNE	Bayesian Nash equilibrium
TN	True network
MN	Misleading network
THA	High-degree attack strategy based on true networks
TLA	Low-degree attack strategy based on true networks
MHA	High-degree attack strategy based on misleading networks
MLA	Low-degree attack strategy based on misleading networks
THD	High-degree defense strategy based on true networks
TLD	Low-degree defense strategy based on true networks
MHD	High-degree defense strategy based on misleading networks
MLD	Low-degree defense strategy based on misleading networks
MD	Strategy set contains MHD and MLD
MA	Strategy set contains MHA and MLA

## Data Availability

Data are contained within the article or Supplementary Materials.

## Supplementary Materials

The following supporting information can be downloaded at: https://www.mdpi.com/article/10.3390/e25010057/s1.
